# Between Nonlinearities, Complexity, and Noises: An Application on Portfolio Selection Using Kernel Principal Component Analysis

**DOI:** 10.3390/e21040376

**Published:** 2019-04-07

**Authors:** Yaohao Peng, Pedro Henrique Melo Albuquerque, Igor Ferreira do Nascimento, João Victor Freitas Machado

**Affiliations:** 1 Campus Universitário Darcy Ribeiro-Brasília, University of Brasilia, Brasilia 70910-900, Brazil; 2 Federal Institute of Piauí, Rua Álvaro Mendes, 94-Centro (Sul), Teresina-PI 64001-270, Brazil

**Keywords:** nonlinearity, regularization, high dimensionality, portfolio allocation, machine learning, covariance estimation, random matrix theory, kernel methods

## Abstract

This paper discusses the effects of introducing nonlinear interactions and noise-filtering to the covariance matrix used in Markowitz’s portfolio allocation model, evaluating the technique’s performances for daily data from seven financial markets between January 2000 and August 2018. We estimated the covariance matrix by applying Kernel functions, and applied filtering following the theoretical distribution of the eigenvalues based on the Random Matrix Theory. The results were compared with the traditional linear Pearson estimator and robust estimation methods for covariance matrices. The results showed that noise-filtering yielded portfolios with significantly larger risk-adjusted profitability than its non-filtered counterpart for almost half of the tested cases. Moreover, we analyzed the improvements and setbacks of the nonlinear approaches over linear ones, discussing in which circumstances the additional complexity of nonlinear features seemed to predominantly add more noise or predictive performance.

## 1. Introduction

Finance can be defined as the research field that studies the management of value—for an arbitrary investor that operates inside the financial market, the value of the assets that he/she chose can be measured in terms of how profitable or risky they are. While individuals tend to pursue potentially larger return rates, often the most profitable options bring along higher levels of uncertainty as well, so that the risk–return relationship induces a trade-off over the preferences of the economic agents, making them seek a combination of assets that offer maximum profitability, as well as minimum risk—an efficient allocation of the resources that generate the most payoff/reward/value.

As pointed out in Miller [1], one of the main milestones in the history of finance was the mean-variance model of Nobel Prize laureate Harry Markowitz, a work regarded as the genesis of the so-called “Modern Portfolio Theory”, in which the optimal portfolio choice was presented as the solution of a simple, constrained optimization problem. Furthermore, Markowitz [2]’s model shows the circumstances in which the levels of risk can be diminished through diversification, as well as the limits of this artifice, represented by a risk that investors can do nothing about and therefore must take when investing in the financial market.

While the relevance of Markowitz [2]’s work is unanimously praised, the best way to estimate its inputs—a vector of expected returns and a covariance matrix—is far from reaching a consensus. While the standard estimators are easy to obtain, recent works like Pavlidis et al. [3] and Hsu et al. [4] argue in favor of the introduction of nonlinear features to boost the predictive power for financial variables over traditional parametric econometric methods, and in which existing novel approaches, such as machine-learning methods, can contribute to better forecasting performances. Additionally, many studies globally have found empirical evidence from real-world financial data that the underlying patterns of financial covariance matrices seem to follow some stylized facts regarding the big proportion of “noise” in comparison to actually useful information, implying that the complexity of the portfolio choice problem could be largely reduced, possibly leading to more parsimonious models that provide better forecasts.

This paper focused on those questions, investigating whether the use of a nonlinear and nonparametric covariance matrix or the application of noise-filtering techniques can indeed help a financial investor to build better portfolios in terms of cumulative return and risk-adjusted measures, namely Sharpe and Sortino ratios. Moreover, we analyzed various robust methods for estimating the covariance matrix, and whether nonlinearities and noise-filtering managed to bring improvements to the portfolios’ performance, which can be useful to the construction of portfolio-building strategies for financial investors. We tested different markets and compared the results, and discussed to which extent the portfolio allocation was done better using Kernel functions and “clean” covariance matrices.

The paper is structured as follows: Section 2 presents the foundations of risk diversification via portfolios, discussing the issues regarding high dimensionality in financial data, motivating the use of high-frequency data, as well as nonlinear predictors, regularization techniques, and the Random Matrix Theory. Section 3 describes the Markowitz [2] portfolio selection model, robust estimators for the covariance matrix, and the Principal Component Analysis for both linear and Kernel covariance matrices. Section 4 provides details on the empirical analysis and describes the collected data and chosen time periods, as well as the performance metrics and statistical tests for the evaluation of the portfolio allocations. Section 5 presents the performance of the obtained portfolios and discusses their implication in view of the financial theory. Finally, Section 6 presents the paper’s conclusions, potential limitations to the proposed methods, and recommendations for future developments.

## 2. Theoretical Background

### 2.1. Portfolio Selection and Risk Management

In financial contexts, “risk” refers to the likelihood of an investment yielding a different return from the expected one [5]; thus, in a broad sense, risk does not necessarily only have regard to unfavorable outcomes (downside risk), but rather includes upside risk as well. Any flotation from the expected value of the return of a financial asset is viewed as a source of uncertainty, or “volatility”, as it is more often called in finance.

A rational investor would seek to optimize his interests at all times, which can be expressed in terms of maximization of his expected return and minimization of his risk. Given that future returns are a random variable, there are many possible measures for its volatility; however, the most common measure for risk is the variance operator (second moment), as used in Markowitz [2]’s Modern Portfolio Theory seminal work, while expected return is measured by the first moment. This is equivalent to assuming that all financial agents follow a mean-variance preference, which is grounded in the microeconomic theory and has implications in the derivation of many important models in finance and asset pricing, such as the CAPM model [6,7,8], for instance.

The assumption of rationality implies that an “efficient” portfolio allocation is a choice of weights w in regard to how much assets you should buy which are available in the market, such that the investor cannot increase his expected return without taking more risk—or, alternatively, how you can decrease his portfolio volatility without taking a lower level of expected return. The curve of the possible efficient portfolio allocations in the risk versus the expected return graph is known as an “efficient frontier”. As shown in Markowitz [2], in order to achieve an efficient portfolio, the investor should diversify his/her choices, picking the assets with the minimal association (measured by covariances), such that the joint risks of the picked assets tend to cancel each other.

Therefore, for a set of assets with identical values for expected return μ and variance $\sigma^{2}$, choosing a convex combination of many of them will yield a portfolio with a volatility value smaller than $\sigma^{2}$, unless all chosen assets have perfect correlation. Such effects of diversification can be seen statistically from the variance of the sum of *p* random variables: $V\left[w_{1}X_{1}+w_{2}X_{2}+\ldots +w_{p}X_{p}\right]=\sum_{i=1}^{p}\sum_{j=1}^{p}w_{i}w_{j}cov\left(X_{i},X_{j}\right)$; since $\sum_{i=1}^{p}w_{i}=1$ (negative-valued weights represent a short selling), the volatility of a generic portfolio $w_{1}x_{1}+w_{2}x_{2}+\ldots +w_{p}x_{p}$ with same-risk assets will always diminish with diversification.

The component of risk which can be diversified, corresponding to the joint volatility between the chosen assets, is known as “idiosyncratic risk”, while the non-diversifiable component of risk, which represents the uncertainties associated to the financial market itself, is known as “systematic risk” or “market risk”. The idiosyncratic risk is specific to a company, industry, market, economy, or country, meaning it can be eliminated by simply investing in different assets (diversification) that will not all be affected in the same way by market events. On the other hand, the market risk is associated with factors that affect all assets’ companies, such as macroeconomic indicators and political scenarios; thus not being specific to a particular company or industry and which cannot be eliminated or reduced through diversification.

Although there are many influential portfolio selection models that arose after Markowitz’s classic work, such as the Treynor-Black model [9], the Black-Litterman model [10], as well as advances in the so-called “Post-Modern Portfolio Theory” [11,12] and machine-learning techniques [13,14,15], Markowitz [2] remains as one of the most influential works in finance and is still widely used as a benchmark for alternative portfolio selection models, due to its mathematical simplicity (uses only a vector of expected returns and a covariance matrix as inputs) and easiness of interpretation. Therefore, we used this model as a baseline to explore the potential improvements that arise with the introduction of nonlinear interactions and covariance matrix filtering through the Random Matrix Theory.

### 2.2. Nonlinearities and Machine Learning in Financial Applications

Buonocore et al. [16] presents two key elements that define the complexity of financial time-series: the multi-scaling property, which refers to the dynamics of the series over time; and the structure of cross-dependence between time-series, which are reflexes of the interactions among the various financial assets and economic agents. In a financial context, one can view those two complexity elements as systematic risk and idiosyncratic risk, respectively, precisely being the two sources of risk that drive the whole motivation for risk diversification via portfolio allocation, as discussed by the Modern Portfolio Theory.

It is well-known that systematic risk cannot be diversified. So, in terms of risk management and portfolio selection, the main issue is to pick assets with minimal idiosyncratic risk, which in turn, naturally, demands a good estimation for the cross-interaction between the assets available in the market, namely the covariance between them.

The non-stationarity of financial time-series is a stylized fact which is well-known by scholars and market practitioners, and this property has relevant implications in forecasting and identifying patterns in financial analysis. Specifically concerning portfolio selection, the non-stationary behavior of stock prices can induce major drawbacks when using the standard linear Pearson correlation estimator in calculating the covariances matrix. Livan et al. [17] provides empirical evidence of the limitations of the traditional linear approach established in Markowitz [2], pointing out that the linear estimator fails to accurately capture the market’s dynamics over time, an issue that is not efficiently solved by simply using a longer historical series. The sensitivity of Markowitz [2]’s model to its inputs is also discussed in Chen and Zhou [18], which incorporates the third and fourth moments (skewness and kurtosis) as additional sources of uncertainty over the variance. Using multi-objective particle swarm optimization, robust efficient portfolios were obtained and shown to improve the expected return in comparison to the traditional mean-variance approach. The relative attractiveness of different robust efficient solutions to different market settings (bullish, steady, and bearish) was also discussed.

Concerning the Dynamical Behavior of Financial Systems, Bonanno et al. [19] proposed a generalization of the Heston model [20], which is defined by two coupled stochastic differential equations (SDEs) representing the log of the price levels and the volatility of financial stocks, and provided a solution for option pricing that incorporated improvements over the classical Black-Scholes model [21] regarding financial stylized facts, such as the skewness of the returns and the excess kurtosis. The extension proposed by Bonanno et al. [19] was the introduction of a random walk with cubic nonlinearity to replace the log-price SDE of Heston’s model. Furthermore, the authors analyzed the statistical properties of escape time as a measure of the stabilizing effect of the noise in the market dynamics. Applying this extended model, Spagnolo and Valenti [22] tested for daily data of 1071 stocks traded at the New York Stock Exchange between 1987 and 1998, finding out that the nonlinear Heston model approximates the probability density distribution on escape times better than the basic geometric Brownian motion model and two well-known volatility models, namely GARCH [23] and the original Heston model [20]. In this way, the introduction of a nonlinear term allowed for a better understanding of a measure of market instability, capturing embedded relationships that linear estimators fail to consider. Similarly, linear estimators for covariance ignore potential associations in higher dimensionality interactions, such that even assets with zero covariance may actually have a very heavy dependence on nonlinear domains.

As discussed in Kühn and Neu [24], the states of a market can be viewed as attractors resulting from the dynamics of nonlinear interactions between the financial variables, such that the introduction of nonlinearities also has potential implications for financial applications, such as risk management and derivatives pricing. For instance, Valenti et al. [25] pointed out that volatility is a monotonic indicator of financial risk, while many large oscillations in a financial market (both upwards and downwards) are preceded by long periods of relatively small levels of volatility in the assets’ returns (the so-called “volatility clustering”). In this sense, the authors proposed the mean first hitting time (defined as the average time until a stock return undergoes a large variation—positive or negative—for the first time) as an indicator of price stability. In contrast with volatility, this measure of stability displays nonmonotonic behavior that exhibits a pattern resembling the Noise Enhanced Stability (NES) phenomenon, observed in a broad class of systems [26,27,28]. Therefore, using the conventional volatility as a measure of risk can lead to its underestimation, which in turn can lead to bad allocations of resources or bad financial managerial decisions.

In light of evidence that not all noisy information of the covariance matrix is due to their non-stationarity behavior [29], many machine-learning methods, such as the Support Vector Machines [30], Gaussian processes [31], and deep learning [32] methods have been discussed in the literature, showing that the introduction of nonlinearities can provide a better display of the complex cross-interactions between the variables and generate better predictions and strategies for the financial markets. Similarly, Almahdi and Yang [33] proposed a portfolio trading algorithm using recurrent reinforcement learning, using the expected maximum drawdown as a downside risk measure and testing for different sets of transaction costs. The authors also proposed an adaptive rebalancing extension, reported to have a quicker reaction to transaction cost variations and which managed to outperform hedge fund benchmarks.

Paiva et al. [34] proposed a fusion approach of a Support Vector Machine and the mean-variance optimization for portfolio selection, testing for data from the Brazilian market and analyzing the effects of brokerage and transactions costs. Petropoulos et al. [35] applied five machine learning algorithms (Support Vector Machine, Random Forest, Deep Artificial Neural Networks, Bayesian Autoregressive Trees, and Naïve Bayes) to build a model for FOREX portfolio management, combining the aforementioned methods in a stacked generalization system. Testing for data from 2001 to 2015 of ten currency pairs, the authors reported the superiority of machine learning models in terms of out-of-sample profitability. Moreover, the paper discussed potential correlations between the individual machine learning models, providing insights concerning their combination to boost the overall predictive power. Chen et al. [36] generalized the idea of diversifying for individual assets for investment and proposed a framework to construct portfolios of investment strategies instead. The authors used genetic algorithms to find the optimal allocation of capital into different strategies. For an overview of the applications of machine learning techniques in portfolio management contexts, see Pareek and Thakkar [37].

Regarding portfolio selection, Chicheportiche and Bouchaud [38] developed a nested factor multivariate model to model the nonlinear interactions in stock returns, as well as the well-known stylized facts and empirically detected copula structures. Testing for the S&P 500 index for three time periods (before, during, and after the financial crisis), the paper showed that the optimal portfolio constructed by the developed model showed a significantly lower out-of-sample risk than the one built using linear Principal Component Analysis, whilst the in-sample risk is practically the same; thus being positive evidence towards the introduction of nonlinearities in portfolio selection and asset allocation models. Montenegro and Albuquerque [39] applied a local Gaussian correlation to model the nonlinear dependence structure of the dynamic relationship between the assets. Using a subset of companies from the S&P 500 Index between 1992 and 2015, the portfolio generated by the nonlinear approach managed to outperform the Markowitz [2] model in more than 60% of the validation bootstrap samples. In regard to the effects of dimensionality reduction on the performance of portfolios generated from mean-variance optimization, Tayalı and Tolun [40] applied Non-negative Matrix Factorization (NMF) and Non-negative Principal Components Analysis (NPCA) for data from three indexes of the Istanbul Stock Market. Optimal portfolios were constructed based on Markowitz [2]’s mean-variance model. Performing backtesting for 300 tangency portfolios (maximum Sharpe Ratio), the authors showed that the portfolios’ efficiency was improved in both NMF and NPCA approaches over the unreduced covariance matrix.

Musmeci et al. [41] incorporated a metric of persistence in the correlation structure between financial assets, and argued that such persistence can be useful for the anticipation of market volatility variations and that they could quickly adapt to them. Testing for daily prices of US and UK stocks between 1997 and 2013, the correlation structure persistence model yielded better forecasts than predictors based exclusively on past volatility. Moreover, the paper discusses the effect of the “curse of dimensionality” that arises in financial data when a large number of assets is considered, an issue that traditional econometric methods often fail to deal with. In this regard, Hsu et al. [4] argues in favor of the use of nonparametric approaches and machine learning methods in traditional financial economics problems, given their better empirical predictive power, as well as providing a broader view of well-established research topics in the finance agenda beyond classic econometrics.

### 2.3. Regularization, Noise Filtering, and Random Matrix Theory

A major setback in introducing nonlinearities is keeping them under control, as they tend to significantly boost the model’s complexity, both in terms of theoretical implications and computational power needed to actually perform the calculations. Nonlinear interactions, besides often being difficult to interpret and apart from a potentially better explanatory power, may bring alongside them a large amount of noisy information, such as an increase in complexity that is not compensated by better forecasts or theoretical insights, but instead which “pollutes” the model by filling it with potentially useless data.

Bearing in mind this setback, the presence of regularization is essential to cope with the complexity levels that come along with high dimensionality and nonlinear interactions, especially in financial applications in which the data-generating processes tend to be highly chaotic. While it is important to introduce new sources of potentially useful information by boosting the model’s complexity, being able to filter that information, discard the noises, and maintain only the “good” information is a big and relevant challenge. Studies like Massara et al. [42] discuss the importance of scalability and information filtering in light of the advent of the “Big Data Era”, in which the boost of data availability and abundance led to the need to efficiently use those data and filter out the redundant ones.

Barfuss et al. [43] emphasized the need for parsimonious models by using information filtering networks, and building sparse-structure models that showed similar predictive performances but much smaller computational processing time in comparison to a state-of-the-art sparse graphical model baseline. Similarly, Torun et al. [44] discussed the eigenfiltering of measurement noise for hedged portfolios, showing that empirically estimated financial correlation matrices contain high levels of intrinsic noise, and proposed several methods for filtering it in risk engineering applications.

In financial contexts, Ban et al. [45] discussed the effects of performance-based regularization in portfolio optimization for mean-variance and mean-conditional Value-at-Risk problems, showing evidence for its superiority towards traditional optimization and regularization methods in terms of diminishing the estimation error and shrinking the model’s overall complexity.

Concerning the effects of high dimensionality in finance, Kozak et al. [46] tested many well-established asset pricing factor models (including CAPM and the Fama-French five-factor model) introducing nonlinear interactions between 50 anomaly characteristics and 80 financial ratios up to the third power (i.e., all cross-interactions between the features of first, second, and third degrees were included as predictors, totaling to models with 1375 and 3400 candidate factors, respectively). In order to shrink the complexity of the model’s high dimensionality, the authors applied dimensionality reduction and regularization techniques considering $\ell_{1}$ and $\ell_{2}$ penalties to increase the model’s sparsity. The results showed that a very small number of principal components were able to capture almost all of the out-of-sample explanatory powers, resulting in a much more parsimonious and easy-to-interpret model; moreover, the introduction of an additional regularized principal component was shown to not hinder the model’s sparsity, but also to not improve predictive performance either.

Depending on the “noisiness” of the data, the estimation of the covariances can be severely hindered, potentially leading to bad portfolio allocation decisions—if the covariances are overestimated, the investor could give up less risky asset combinations, or accept a lesser expected profitability; if the covariances are underestimated, the investor would be bearing a higher risk than the level he was willing to accept, and his portfolio choice could be non-optimal in terms of risk and return. Livan et al. [17] discussed the impacts of measurement noises on correlation estimates and the desirability of filtering and regularization techniques to diminish the noises in empirically observed correlation matrices.

A popular approach for the noise elimination of financial correlation matrices is the Random Matrix Theory, which studies the properties of matrix-form random variables—in particular, the density and behavior of eigenvalues. Its applications cover many of the fields of knowledge of recent years, such as statistical physics, dynamic systems, optimal control, and multivariate analysis.

Regarding applications in quantitative finance, Laloux et al. [47] compared the empirical eigenvalues density of major stock market data with their theoretical prediction, assuming that the covariance matrix was random following a Wishart distribution (If a vector of random matrix variables follows a multivariate Gaussian distribution, then its Sample covariance matrix will follow a Wishart distribution [48]).The results showed that over 94% of the eigenvalues fell within the theoretical bounds (defined in Edelman [48]), implying that less than 6% of the eigenvalues contain actually useful information; moreover, the largest eigenvalue is significantly higher than the theoretical upper bound, which is evidence that the covariance matrix estimated via Markowitz is composed of few very informative principal components and many low-valued eigenvalues dominated by noise. Nobi et al. [49] tested for the daily data of 20 global financial indexes from 2006 to 2011 and also found out that most eigenvalues fell into the theoretical range, suggesting a high presence of noises and few eigenvectors with very highly relevant information; particularly, this effect was even more prominent during a financial crisis. Although studies like El Alaoui [50] found a larger percentage of informative eigenvalues, the reported results show that the wide majority of principal components is still dominated by noisy information.

Plerou et al. [51] found similar results, concluding that the top eigenvalues of the covariance matrices were stable in time and the distribution of their eigenvector components displayed systematic deviations from the Random Matrix Theory predicted thresholds. Furthermore, the paper pointed out that the top eigenvalues corresponded to an influence common to all stocks, representing the market’s systematic risk, and their respective eigenvectors showed a prominent presence of central business sectors.

Sensoy et al. [52] tested 87 benchmark financial indexes between 2009 and 2012, and also observed that the largest eigenvalue was more than 14 times larger than the Random Matrix Theory theoretical upper bound, while only less than 7% of the eigenvalues were larger than this threshold. Moreover, the paper identifies “central” elements that define the “global financial market” and analyzes the effects of the 2008 financial crisis in its volatility and correlation levels, concluding that the global market’s dependence level generally increased after the crisis, thus making diversification less effective. Many other studies identified similar patterns in different financial markets and different time periods [53,54], evidencing the high levels of noise in correlation matrices and the relevance of filtering such noise for financial analysis. The effects of the covariance matrix cleaning using Random Matrix Theory in an emerging market was discussed in Eterovic and Eterovic [55], which analyzed 83 stocks from the Chilean financial market between 2000 and 2011 and found out that the efficiency of portfolios generated using Markowitz [2]’s model were largely improved.

Analogously, Eterovic [56] analyzed the effects of covariance matrix filtering through the Random Matrix Theory using data from the stocks of the FTSE 100 Index between 2000 and 2012, confirming the distribution pattern of the eigenvalues of the covariance matrix, with the majority of principal components inside the bounds of the Marčenko-Pastur distribution, while the top eigenvalue was much larger than the remaining ones; in particular, the discrepancy of the top eigenvalue was even larger during the Crisis period. Moreover, Eterovic [56] also found out that the performance improvement of the portfolios generated by a filtered covariance matrix filtering over a non-filtered one was strongly significant, evidencing the ability of the filtered covariance matrix to adapt to sudden volatility peaks.

Bouchaud and Potters [57] summarized the potential applications of the Random Matrix Theory in financial problems, focusing on the cleaning of financial correlation matrices and the asymptotic behavior of its eigenvalues, whose density was enunciated in Marčenko and Pastur [58]—and especially the largest one, which was described by the Tracy-Widom distribution [59]. The paper presents an empirical application using daily data of US stocks between 1993 and 2008, observing the correlation matrix of the 500 most liquid stocks in a sliding window of 1000 days with an interval of 100 days each, yielding 26 sample eigenvalue distributions. On average, the largest eigenvalue represents 21% of the sum of all eigenvalues. This is a stylized fact regarding the spectral properties of financial correlation matrices, as discussed in Akemann et al. [60]. Similar results were found in Conlon et al. [61], which analyzes the effects of “cleaning” the covariance matrix on better predictions of the risk of a portfolio, which may aid the investors to pick the best combination of hedge funds to avoid risk.

In financial applications, the covariance matrix is also important in multi-stage optimization problems, whose dimensionality often grows exponentially as the number of stages, financial assets or risk factor increase, thus demanding approximations using simulated scenarios to circumvent the curse of dimensionality [62]. In this framework, an important requirement for the simulated scenarios is the absence of arbitrage opportunities, a condition which can be incorporated through resampling or increasing the number of scenarios [63]. Alternatively, [64] defined three classes for arbitrage propensity and suggested a transformation on the covariance matrix’s Cholesky decomposition that avoids the possibility of arbitrage in scenarios where it could theoretically exist. In this way, the application of the Random Matrix Theory on this method can improve the simulated scenarios in stochastic optimization problems, and consequently improve the quality of risk measurement and asset allocation decision-making.

Burda et al. [65] provided a mathematical derivation of the relationship between the sample correlation matrix calculated using the conventional Pearson estimates with its population counterpart, discussing how the dependency structure of the spectral moments can be applied to filter out the noisy eigenvalues of the correlation matrix’s spectrum. In fact, a reasonable choice of a 500×500 covariance matrix (like using the S&P 500 data for portfolio selection) induces a very high level of noise in addition to the signal that comes from the eigenvalues of the population covariance matrix; Laloux et al. [66] used daily data of the S&P 500 between 1991 and 1996, and found out that the covariance matrix estimated by the classical Markowitz model highly underestimates the portfolio risks for a second time period (approximately three times lower than the actual values), a difference that is significantly lower for a cleaned correlation matrix, evidencing the high level of noise and the instability of the market dependency structure over time.

In view of the importance of controlling the complexity introduced alongside nonlinearities, in this paper we sought to verify whether the stylized behavior of the top eigenvalues persists after introducing nonlinearities into the covariance matrix, as well as the effect of cleaning the matrix’s noises in the portfolio profitability and consistency over time, in order to obtain insights regarding the cost–benefit relationship between using higher degrees of nonlinearity to estimate the covariance between financial assets and the out-of-sample performance of the resulting portfolios.

## 3. Method

### 3.1. Mean-Variance Portfolio Optimization

Let $a_{1},a_{2},\ldots ,a_{p}$ be the *p* available financial assets and $r_{a_{i}}$ be the return vector of the *i*-th asset $a_{i}$, where the expected return vector and the covariance matrix are defined, respectively, as $\mu =\left(\mu_{1},\mu_{2},\ldots ,\mu_{p}\right)=\left(E\left[r_{a_{1}}\right],E\left[r_{a_{2}}\right],\ldots ,E\left[r_{a_{p}}\right]\right)$ and $\Sigma =(\sigma_{ij}),\;i,j=1,2,\ldots ,p$, with $\sigma_{ij}=cov\left(r_{a_{i}},r_{a_{j}}\right)$. Markowitz [2]’s mean-variance portfolio optimization is basically a quadratic programming constrained optimization problem whose optimal solution $w={\left(w_{1},w_{2},...,w_{p}\right)}^{T},\;\sum_{i=1}^{p}w_{i}=1$ represents the weights allocated to each one of the *p* assets, such that the portfolio $P=w_{1}a_{1}+w_{2}a_{2}+\ldots +w_{p}a_{p}$. Algebraically, the expected return and the variance of the resulting portfolio P are:

$$\begin{array}{ccc} E[P]= & \sum_{i=1}^{p}w_{i}E\left[r_{a_{i}}\right] & =\mu^{T}w\in R\\ V[P]= & \sum_{i=1}^{p}\sum_{j=1}^{p}w_{i}w_{j}cov\left(r_{a_{i}},r_{a_{j}}\right) & =w^{T}\Sigma w\geq 0 \end{array}$$

With the non-allowance of a short selling constraint, the quadratic optimization problem is defined as:

(1) $$\begin{array}{cc} \mathrm{Minimize}: & \frac{1}{2}w^{T}\Sigma w\\ \mathrm{Subject}\;\mathrm{to}: & \mu^{T}w=R,w^{T}1=1,w>0 \end{array}$$

which yields the weights that give away the less risky portfolio that provides an expected return equal to *R*; therefore, the portfolio P that lies on the efficient frontier for E[P]=R. The dual form of this problem has an analogous interpretation—instead of minimizing the risk at a given level of expected return, it maximizes the expected return given a certain level of tolerated risk.

Markowitz [2]’s model is very intuitive, easy to interpret, and enjoys huge popularity to this very day, making it one of the main baseline models for portfolio selection. Moreover, it has only two inputs which are fairly easy to be estimated. Nevertheless, there are many different ways of doing so, which was the motivation of many studies to tackle this question, proposing alternative ways to estimate those inputs to find potentially better portfolios. The famous Black and Litterman [10] model, for example, proposes a way to estimate the expected returns vector based on the combination of market equilibrium and the expectations of the investors operating in that market. In this paper, we focus on alternative ways to estimate the covariance matrix, and whether features like nonlinearities (Kernel functions) and noise filtering (Random Matrix Theory) can generate more profitable portfolio allocations.

### 3.2. Covariance Matrices

While Pearson’s covariance estimator is consistent, studies like Huo et al. [67] pointed out that the estimates can be heavily influenced by outliers, which in turn leads to potentially suboptimal portfolio allocations. In this regard, the authors analyzed the effect of introducing robust estimation of covariance matrices, with the results of the empirical experiments showing that the use of robust covariance matrices generated portfolios with larger profitabilities. Zhu et al. [68] found similar results, proposing a high-dimensional covariance estimator less prone to outliers and leading to more well-diversified portfolios, often with a higher alpha.

Bearing in mind the aforementioned findings of the literature, we tested KPCA and the noise filtering to many robust covariance estimators as well, in order to further investigate the effectiveness of nonlinearities introduction and the elimination of noisy eigenvalues to the portfolio’s performance. Furthermore, we intended to check the relative effects of said improvements to Pearson and robust covariance matrices, and whether robust estimators remained superior under such conditions.

In addition to the Pearson covariance matrix $\Sigma =\frac{1}{T}\sum_{i=1}^{p}x_{i}x_{i}^{T}$, where $x_{i}$ is the return vector (centered in zero) of the *i*-th asset and *T* is the number of in-sample time periods, in this paper we considered four robust covariance estimators: the minimum covariance determinant (henceforth MCD) method [69], as estimated by the FASTMCD algorithm [70]; the Reweighted MCD, following [71]’s algorithm; and the Orthogonalized Gnanadesikan-Kettenring (henceforth OGK) pairwise estimator [72], following the algorithm of [73].

The MCD method aims to find observations whose sample covariance has a minimum determinant, thus being less sensitive to non-persistent extreme events, such as an abrupt oscillation of price levels that briefly come back to normal. Cator and Lopuhaä [74] demonstrated some statistical properties of this estimator, such as consistency and asymptotic convergence to the Gaussian distribution. The reweighted MCD estimator follows a similar idea, assigning weights to each observation and computing the covariance estimates based on the observations within a confidence interval, making the estimates even less sensitive to outliers and noisy datasets, as well as boosting the finite-sample efficiency of the estimator, as discussed in Croux and Haesbroeck [75]. Finally, the OGK approach takes univariate robust estimators of location and scale, constructing a covariance matrix based on those estimates and replacing the eigenvalues of that matrix with “robust variances”, which are updated sequentially by weights based on a confidence interval cutoff.

### 3.3. Principal Component Analysis

Principal component analysis (henceforth PCA) is a technique for dimensionality reduction introduced by [76] which seeks to extract the important information from the data and to express this information as a set of new orthogonal variables called principal components, given that the independent variables of a dataset are generally correlated in some way. Each of these principal components is a linear combination of the set of variables in which the coefficients show the importance of the variable to the component. By definition, the sum of all eigenvalues is equal to the total variance, as they represent an amount of observed information; therefore, each eigenvalue represents the variation explained of the *i*-th principal component $PC_{i}$, such that their values reflect the proportion of information maintained in the respective eigenvector, and thus are used to determine how many factors should be retained.

In a scenario with *p* independent variables, if it is assumed that the eigenvalues’ distribution is uniform, then each eigenvalue would contribute to $\frac{1}{p}$ of the model’s overall explanatory power. Therefore, taking a number k<p of principal components that are able to explain more than $\frac{k}{p}$ of the total variance can be regarded as a “gain” in terms of useful information retaining and noise elimination. In the portfolio selection context, Kim and Jeong [77] used PCA to decompose the correlation matrix of 135 stocks traded on the New York Stock Exchange (NYSE). Typically, the largest eigenvalue is considered to represent a market-wide effect that influences all stocks [78,79,80,81].

Consider Σ as a covariance matrix associated with the random vector $X=[X_{1},X_{2}\ldots ,X_{p}]$ with eigenvalues $\lambda_{1}\geq \lambda_{2}\ldots \geq \lambda_{p}\geq 0$, where the rotation of the axis in $R^{p}$ yields the linear combinations:

$$\begin{array}{c} Y_{1}=q_{1}^{T}X=q_{11}X_{1}+q_{12}X_{2}+\ldots +q_{1p}X_{p}\\ Y_{2}=q_{2}^{T}X=q_{21}X_{2}+q_{22}X_{2}+\ldots +q_{2p}X_{p}\\ \vdots \;\;\;\;\;\;\;\\ Y_{p}=q_{p}^{T}X=q_{p1}X_{1}+q_{p2}X_{2}+\ldots +q_{pp}X_{p}\\ \mathrm{or}\;\;\;\;\;\\ Y=Q^{T}X\;\;\;\; \end{array}$$

where $Q_{i}$ are the eigenvectors from Σ. Thus, the first principal component $Y_{1}$ is the projection in the direction in which the variance of the projection is maximized. So, we obtained $Y_{1},Y_{2}\ldots Y_{p}$ orthonormal vectors with maximum variability.

To obtain the associated eigenvectors, we solved for det(Σ−λI)=0 to obtain the diagonal matrix composed by the eigenvalues. The variance of the $i_{th}$ principal component of Σ is equal to its *i*-th eigenvalue $\lambda_{i}$. By construction, the principal component are pairwise orthogonal—that is, the covariance between the eigenvectors is $cov(Q_{i}X,Q_{j}X)=0,\;i\neq j$. Algebraically, the *i*-th principal component $Y_{i}$ can be obtained by solving the following expression for $a_{i}$ [82]:

(2) $$\max_{q_{i}}\left\{\frac{q_{i}\sum_{i=1}^{p}q_{i}}{q_{i}^{T}q_{i}}\;\;cov\left(Y_{i},Y_{j}\right)=0,\forall \;0<j<i\right\}$$

In the field of dimensionality reduction, the interest in entropy, the entropy-based distance metric, has been investigated, where [83] developed kernel entropy component analysis (KECA) for data transformation and dimensionality reduction, an extension of PCA mixture entropy and n dimensionality decomposition. [84] shows that by using kernel entropy component analysis in an application on face recognition algorithm based on Renyi entropy component, certain eigenvalues and the corresponding eigenvectors will contribute more to the entropy estimate than others, since the terms depend on different eigenvalues and eigenvectors.

### 3.4. Kernel Principal Component Analysis and Random Matrix Theory

Let X be a T×p matrix, *T* being the observations, *p* the variables, and Σ the covariance matrix p×p. The spectral decomposition of Σ is given by:

λQ=ΣQ

being λ≥0 the eigenvalues and Q the eigenvectors.

If the values of matrix *X* are random normalized values generated by a Gaussian distribution, then if T→∞ and p→∞ where $\Psi =\frac{T}{p}\geq 1$ the eigenvalues of matrix Σ result in the following probability density function [61]:

(3) $$p\left(\lambda \right)=\frac{\Psi }{2\pi }\frac{\sqrt{\left(\lambda_{max}-\lambda \right)\left(\lambda -\lambda_{min}\right)}}{\lambda }$$

where $\lambda_{max}$ and $\lambda_{min}$ are the bound given by:

(4) $$\lambda_{min}^{max}=\left(1+\frac{1}{\Psi }\pm 2\sqrt{\frac{1}{\Psi }}\right)$$

This result basically states that the eigenvalues of a purely random matrix based on distribution (3) tend to fall inside the theoretical boundaries; thus, eigenvalues larger than the upper bound are expected to contain useful information concerning an arbitrary matrix, whilst the noisy information is dispersed into the other eigenvalues, whose behavior is similar to the eigenvalues of a matrix with no information whatsoever.

There are many applications of the Random Matrix Theory (RMT) in the financial context. Ref. [85] used RMT to reduce the noise into data before to model the covariance matrix of assets on Asset Pricing Theory Models by using the Bayesian approach. The posteriori distribution was adjusted by Wishart Distribution using MCMC methods.

The procedures proposed by RMT for dispersion matrices noise filter in a finances context require careful use. The reasons are due to the “stylized facts” present in this type of data as logarithmic transformations in the attempt for symmetric distributions of returns and the presence of extreme values. The work of [86] deals with these problems and uses Tyler’s robust M-estimator [87] to estimate the dispersion matrix to then identify the non-random part with the relevant information via RMT using [58] bounds.

The covariance matrix Σ can be factored as:

(5) $$\Sigma =Q\Lambda Q^{-1}$$

where Λ is a diagonal matrix composed by *p* eigenvalues $\lambda_{i}\geq 0,\;i=1,2,\ldots ,p$ and each one of the *p* columns of Q, $q_{i},\;i=1,2,\ldots ,p$, are the eigenvectors associated with the *i*-th eigenvector $\lambda_{i}$. The idea is to perform the decomposition of Σ following Equation (5) and to filter out the eigenvalues which fall inside the boundaries postulated in Equation (4) and reconstruct Σ by multiplying back the filtered eigenvalue matrix to the eigenvector matrices, and then using the filtered matrix as input to Markowitz [2]’s model.

Eigenvalues smaller than the upper bound of Equation (4) were considered as “noisy eigenvalues”, while eigenvalues larger than the upper bound were considered “non-noisy”. For the eigenvalue matrix filtering, we maintained all non-noisy eigenvalues and replaced all the remaining noisy ones by their average in order to preserve the stability (positive-definitiveness) and keep a fixed sum for the matrix’s trace, following Sharifi et al. [88] and Conlon et al. [61].

For eigenvalue matrix filtering, we maintained all non-noisy eigenvalues in Λ and replaced all the remaining noisy ones $\lambda_{i}^{noise}$ by their average (${\bar{\lambda }}_{i}^{noise}$):

$${\bar{\lambda }}_{i}^{noise}=\sum_{i=1}^{\Omega \in noise}\frac{\lambda_{i}^{noise}}{\#\Omega \in noise}$$

After the filtering process, we multiplied back the filtered eigenvalue matrix to yield the “clean” covariance matrix:

(6) $$\Sigma^{*}=Q\Lambda^{*}Q^{-1}$$

where $\Lambda^{*}$ is a diagonal matrix composed of the cleaned eigenvalues.

The nonlinear estimation of the covariance matrix was achieved by means of a Kernel function, defined as:

(7) $$\kappa \left(x_{i},x_{j}\right)=\varphi^{T}\left(x_{i}\right)\cdot \varphi \left(x_{j}\right)\in R,\;i,j=1,2,\ldots ,p$$

where $\varphi :R^{p}\Rightarrow R^{q},p<q$ transforms the original data to a higher dimension, which can even be infinite, and the use of the kernel function prevents the need to explicitly compute the functional form of φ(x); instead, κ computes the inner product of φ. This is known as the *kernel trick*. The use of the Kernel function can circumvent the problem of high dimensionality induced by φ(x) without the need to explicitly compute its functional form; instead, all nonlinear interactions between the original variables are synthesized in a real scalar. Since the inner product is a similarity measure in Hilbert spaces, the Kernel function can be seen as a way to measure the “margin” between the classes in high (or even infinite) dimensional spaces.

The following application of the Kernel function to the linearly estimated covariance matrix:

(8) $$\Sigma =\frac{1}{T}\sum_{i=1}^{p}x_{i}x_{i}^{T}$$

allows one to introduce a high number of nonlinear interactions in the original data and transform Σ into a Kernel covariance matrix:

(9) $$\Sigma_{\kappa }=\frac{1}{T}\sum_{i=1}^{p}\varphi \left(x_{i}\right)\varphi^{T}\left(x_{i}\right)$$

In this paper, we tested the polynomial and Gaussian Kernels as κ. Both Kernels are widely used functions in the machine learning literature. The polynomial Kernel:

(10) $$\kappa \left(x_{i},x_{j}\right)={\left[\left(x_{i}\cdot x_{j}\right)+d\right]}^{q},d\in R,q\in N^{+}$$

has a concise functional form, and is able to incorporate all cross-interactions between the explanatory variables that generate monomials with a degree less than or equal to a predefined *q*. This paper considered polynomial Kernels of degrees 2, 3, and 4 (q=2,3,4). Note that the polynomial Kernel with q=1 and d=0 precisely yields the Pearson linear covariance matrix, so the polynomial Kernel covariance matrix is indeed a more general case of the former.

The Gaussian Kernel is the generalization of the polynomial Kernel for q→∞, and is one of the most widely used Kernels in machine learning literature. It enjoys huge popularity in various knowledge fields since this function is able to induce an infinite dimensional feature space while depending on only one scattering parameter σ. The expression of the Gaussian Kernel is given by:

(11) $$\kappa \left(x_{i},x_{j}\right)=\exp\left(-\frac{x_{i}-x_{j}^{2}}{2\sigma^{2}}\right),\sigma >0$$

The Kernel Principal Component Analysis (henceforth KPCA) is an extension of the linear PCA applied to the Kernel covariance matrix. Basically, the diagonalization problem returns linear combinations from the Kernel function’s feature space $R^{q}$, instead of the original input space $R^{p}$ with the original variables. By performing the spectral decomposition in the Kernel covariance matrix:

(12) $$\Sigma_{\kappa_{\left(\mathrm{pxp}\right)}}=Q\Lambda_{\kappa }Q^{-1}$$

and extracting the largest eigenvalues of the Kernel covariance eigenvalue matrix $\Lambda_{\kappa }$, we obtained the filtered Kernel covariance eigenvalue matrix $\Lambda_{\kappa }^{*}$, which was then used to reconstruct the filtered Kernel covariance matrix:

(13) $$\Sigma_{\kappa_{\left(\mathrm{pxp}\right)}}^{*}=Q\Lambda_{\kappa }^{*}Q^{-1}$$

Finally, $\Sigma_{\kappa }^{*}$ was used as an input for the Markowitz portfolio optimization model, and the resultant portfolio’s profitability was compared to the portfolio generated by the linear covariance matrix and other aforementioned robust estimation methods, as well as their filtered counterparts. The analysis was reiterated for data from seven different markets, and the results are discussed in Section 5.

The pseudocode of our proposed approach is displayed as follows:

1. Estimate Σ for training set data;
2. Perform spectral decomposition of Σ: $\Sigma =Q\Lambda Q^{-1}$;
3. From the eigenvalues matrix Λ, identify the noisy eigenvalues $\lambda_{i}^{noise}$ based on the Random Matrix Theory upper bound;
4. Replace all noisy by their average: ${\bar{\lambda }}_{i}^{noise}$ to obtain the filtered eigenvalue matrix $\Lambda^{*}$;
5. Build the filtered covariance matrix $Q\Lambda^{*}Q^{-1}$;
6. Use the filtered covariance matrix as input for Markowitz model and get the optimal portfolio weights from in-sample data;
7. Apply the in-sample optimal portfolio weights for out-of-sample data and obtain performance measures.

The above procedure was repeated for all seven datasets (NASDAQ 100, CAC 40, DAX-30, FTSE 100, NIKKEI 225, IBOVESPA, SSE 180). For Step **1** (estimation method of the covariance matrix), we applied eight different methods, namely: linear (Pearson), minimum covariance determinant (MCD), reweighted minimum covariance determinant (RMCD), Orthogonalized Gnanadesikan-Kettenring (OGK), Polynomial Kernel functions of degree 2 (K_POLY2), degree 3 (K_POLY3) and degree 4 (K_POLY4), and the Gaussian Kernel function (K_GAUSS).

## 4. Empirical Analysis

### 4.1. Performance Measures

The trade-off between risk and return has long been well-known in the finance literature, where higher expected return generally implies a greater level of risk, which motivates the importance of considering risk-adjusted measures of performance. Therefore, it is not sufficient to view a portfolio’s attractiveness only in terms of the cumulative returns that it offers, but instead, whether the return compensates for the level of risk that the allocation exposes the investor to. The Sharpe ratio [89] provides a simple way to do so.

Let P be a portfolio composed by a linear combination between assets whose expected return vector is r, considering w as the weight vector of P and $r_{f_{t}}$ as the risk-free rate at time *t*. Defining the mean excess return over the risk-free asset of P along the *N* out-of-sample time periods as:

(14) $${\bar{\mu }}_{P}=\frac{1}{N}\sum_{t=1}^{N}w_{t}^{T}r_{t}-r_{f_{t}}$$

and defining the sample standard deviation of portfolio P as:

(15) $$\sigma_{P}=\sqrt{\frac{1}{N-1}\sum_{t=1}^{N}{\left(w_{t}^{T}r_{t}-r_{f_{t}}-{\bar{\mu }}_{P}\right)}^{2}}$$

The Sharpe ratio of portfolio P is given by:

(16) $$ShR_{P}=\frac{\bar{\mu }}{\sigma_{P}}$$

While the Sharpe ratio gives a risk-adjusted performance measure for a portfolio and allows direct comparison between different allocations, it has the limitation of considering both the upside and the downside risks. That is, the uncertainty of profits is penalized in the Sharpe ratio expression, even though it is positive for an investor. Therefore, as discussed in works like Patton and Sheppard [90] and Farago and Tédongap [91], the decomposition of risk in “good variance” and “bad variance” can provide better asset allocation and volatility estimation, thus leading to better investment and risk management decisions. Therefore, instead of using the conventional standard deviation, which considers both methods of variance, Sortino and Price [92] proposed an alternative performance measure that became known as the Sortino ratio, which balances the mean excess return only by the downside deviation. The Sortino ratio for portfolio P is given by:

(17) $$SoR_{P}=\frac{\bar{\mu }}{\sigma_{P}^{-}}$$

where $\sigma_{P}^{-}$ is the downside deviation:

(18) $$\sigma_{P}^{-}=\sqrt{\frac{1}{N-1}\sum_{t=1}^{N}min(0,{\left(w_{t}^{T}r_{t}-r_{f_{t}}-{\bar{\mu }}_{P}\right)}^{2})}$$

Note that the downside deviation represents the standard deviation of negative portfolio returns, thus measuring only the “bad” side of volatility; for periods that the portfolio yields a better return than the mean excess return over the risk-free asset, this upside deviation is not accounted for by the Sortino ratio.

Furthermore, we tested the statistical significance of the covariance matrix filtering improvement on the portfolio’s performance. That is, instead of just comparing the values of the ratios, we tested to which extent the superiority of the noise-filtering approach was statistically significant. For each model and each analyzed market, we compared the Sharpe and Sortino ratios of the non-filtered covariance matrices with their respective filtered counterparts using Student’s *t* tests. The null and alternative hypothesis are defined as follows:

(19) $$\left\{\begin{array}{cc} H_{0}: & ShR_{non-filtered}=ShR_{filtered}\\ H_{A}: & ShR_{non-filtered}<ShR_{filtered} \end{array}\right.$$

(20) $$\left\{\begin{array}{cc} H_{0}: & SoR_{non-filtered}=SoR_{filtered}\\ H_{A}: & SoR_{non-filtered}<SoR_{filtered} \end{array}\right.$$

Rejection of both null hypotheses implies that the Sharpe/Sortino ratio of the portfolio generated using the filtered covariance matrix is statistically larger than the portfolio yielded by the non-filtered matrix. The *p*-values for the hypothesis tests are displayed in Table 1, Table 2, Table 3, Table 4, Table 5, Table 6 and Table 7.

### 4.2. Data

For the empirical application, we used data from seven markets—namely, the United States, United Kingdom, France, Germany, Japan, China, and Brazil; the chosen financial indexes representing each market were, respectively, NASDAQ-100, FTSE 100, CAC 40, DAX-30, NIKKEI 225, SSE 180 and Bovespa. We collected the daily return of the financial assets that composed those indexes during all time periods between 1 January 2000 and 16 August 2018, totaling 4858 observations for each asset. The data was collected from the Bloomberg terminal. The daily excess market return over the risk-free rate was collected from Kenneth R. French’s data library (http://mba.tuck.dartmouth.edu/pages/faculty/ken.french/data_library.html).

We split the datasets into two mutually exclusive subsets: we allocated the observations between 1 January 2000 and 3 November 2015 (85% of the whole dataset, 4131 observations) for the training (in-sample) subset and the observations between 4 November 2015 and 16 August 2018 (the remaining 15%, 727 observations) for the test (out-of-sample) subset. For each financial market and each covariance matrix method, we estimated the optimal portfolio for the training subset and applied the optimal weights for the test subset data. The cumulative return of the portfolio in the out-of-sample periods, their Sharpe and Sortino ratios, information regarding the non-noisy eigenvalues and *p*-values of tests (19) and (20) are displayed in Table 1, Table 2, Table 3, Table 4, Table 5, Table 6 and Table 7.

## 5. Results and Discussion

**Figure 1:**
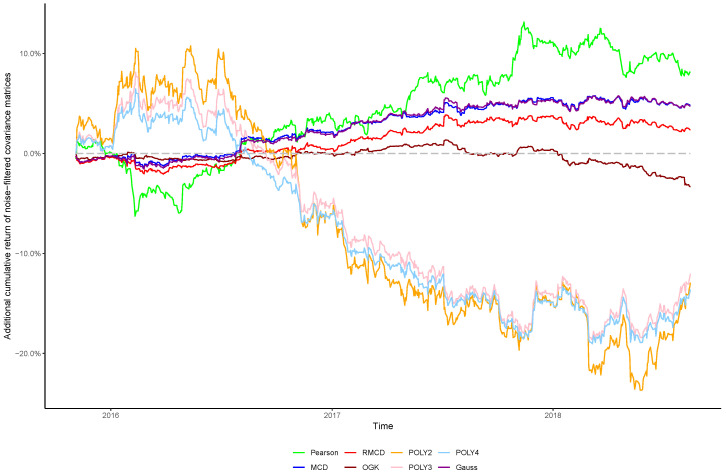
Cumulative return improvement of noise-filtered covariance matrices over non-filtered ones for assets of NASDAQ-100 Index during the out-of-sample period.

**Figure 2:**
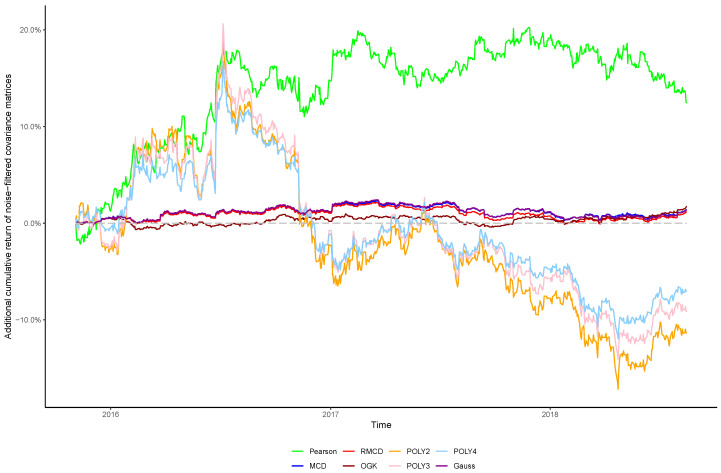
Cumulative return improvement of noise-filtered covariance matrices over non-filtered ones for assets of FTSE 100 Index during the out-of-sample period.

**Figure 3:**
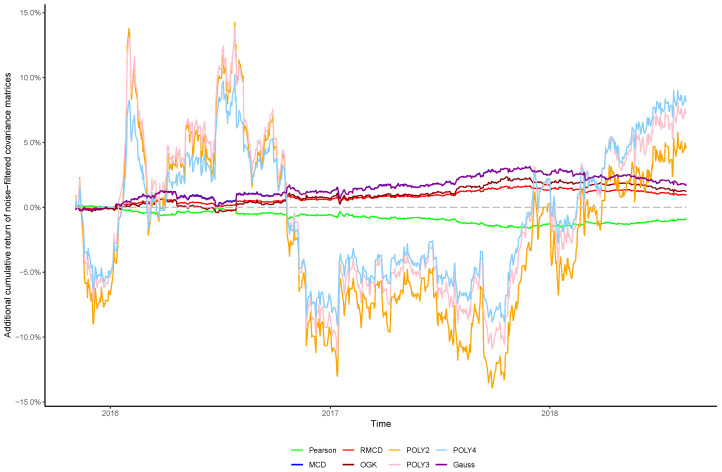
Cumulative return improvement of noise-filtered covariance matrices over non-filtered ones for assets of CAC 40 Index during the out-of-sample period.

**Figure 4:**
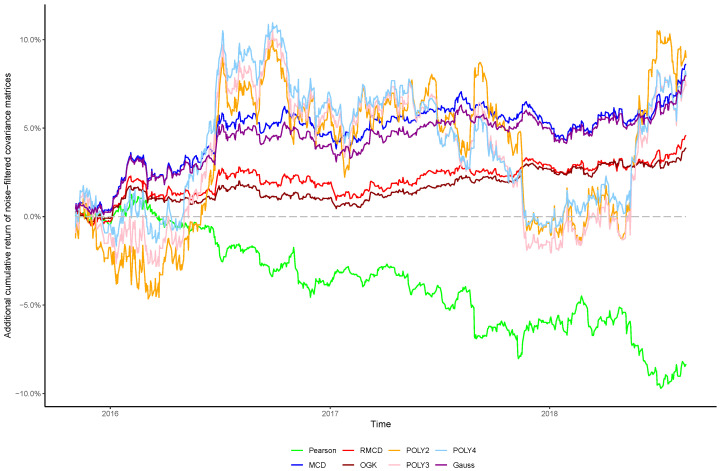
Cumulative return improvement of noise-filtered covariance matrices over non-filtered ones for assets of the DAX-30 Index during the out-of-sample period.

**Figure 5:**
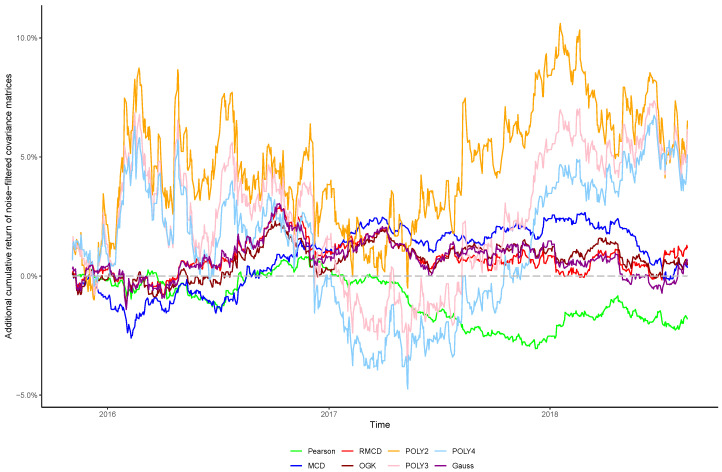
Cumulative return improvement of noise-filtered covariance matrices over non-filtered ones for assets of the NIKKEI 225 Index during the out-of-sample period.

**Figure 6:**
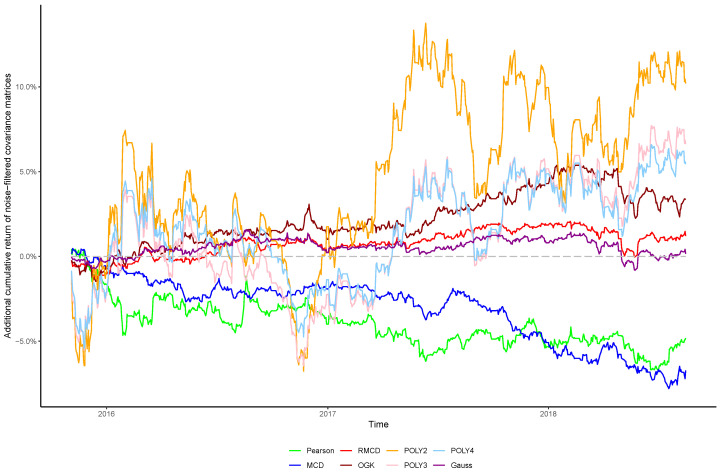
Cumulative return improvement of noise-filtered covariance matrices over non-filtered ones for assets of the SSE 180 Index during the out-of-sample period.

**Figure 7:**
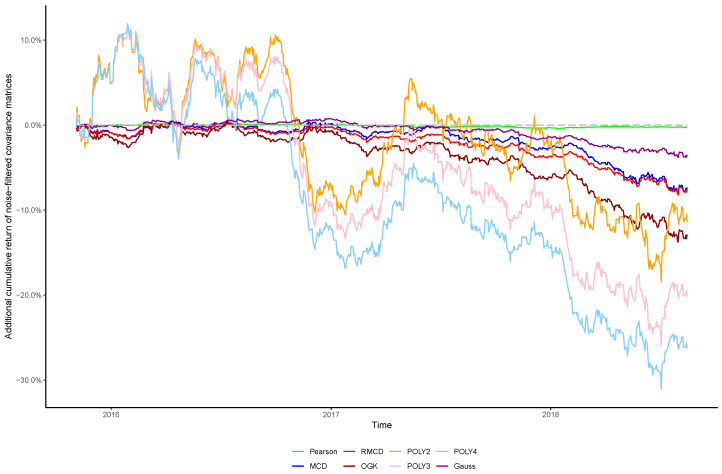
Cumulative return improvement of noise-filtered covariance matrices over non-filtered ones for assets of Bovespa 100 Index during the out-of-sample period.

The cumulative returns and risk-adjusted performance metrics are presented in Table 1, Table 2, Table 3, Table 4, Table 5, Table 6 and Table 7, as well as information regarding the non-noisy eigenvalues and the *p*-values of the hypothesis tests. Figure 1, Figure 2, Figure 3, Figure 4, Figure 5, Figure 6 and Figure 7 show the improvement of filtered covariance matrices over their non-filtered counterparts for each market and estimation method. The results are summarized as follows:

For the non-filtered covariance matrices, the overall performance of the linear Pearson estimates was better than robust estimation methods in most markets, although it was outperformed by all three robust methods (MCD, RMCD, and OGK) for the CAC and SSE indexes. In comparison to the nonlinear covariance matrices induced by the application of Kernel functions, the linear approaches performed better in four out of the seven analyzed markets (CAC, DAX, NIKKEI, and SSE), although in the other three markets the nonlinear models performed better by a fairly large margin. Between the robust estimators, the performance results were similar, slightly favoring the OGK approach. Amongst the nonlinear models, the Gaussian Kernel generally performed worse than the polynomial Kernels—an expected result, as the Gaussian Kernel incorporates polynomial interactions that effectively tends to infinity-degree, which naturally inserts a large amount of noisy information; the only market where the Gaussian Kernel performed notably better was the Brazilian one, which is considered to be an “emerging economy” and a less efficient market compared to the United States or Europe; even though Brazil is the leading market in Latin America, this market’s liquidity, transaction flows, and informational efficiency are quite smaller compared to major financial markets (For a broad discussion about the dynamics of financial markets of emerging economies, see Karolyi [93]). Therefore, it is to be expected that such a market contains more levels of “noise”, such that a function that incorporates a wider range of nonlinear interactions tend to perform better.

As for the filtered covariance matrices, the Pearson estimator and the robust estimators showed similar results, with no major overall differences in profitability or risk-adjusted measures—Pearson performed worst than MCD, RMCD, and OGK for NASDAQ and better for FTSE and DAX. In comparison to MCD and OGK, the RMCD showed slightly better performance. Similarly to the non-filtered cases, the polynomial Kernels yielded generally better portfolios in most markets. Concerning the Gaussian Kernel, even though its filtered covariance matrix performed particularly well for FTSE and Bovespa, it showed very bad results for the German and Chinese markets, suggesting that an excessive introduction of nonlinearities may bring along more costs than improvements. Nevertheless, during the out-of-sample periods, the British and Brazilian markets underwent exogenous events—namely the effects of the “Brexit” referendum for the United Kingdom and the advancements of the “Car Wash” (*Lava Jato*) operation, which led to events like the prison of Eduardo Cunha (former President of the Chamber of Deputies) in October 2016; and Luis Inácio da Silva (former President of Brazil) in April 2018—that may have affected their respective systematic levels of risk and profitability, potentially compromising the market as a whole. In this sense, the fact that the Gaussian Kernel-filtered covariance matrices in those markets performed better than the polynomial Kernels is evidence that the additional levels of “complexity” in those markets may demand the introduction of more complex nonlinear interactions to make good portfolio allocations. These results are also consistent with the finding of Sandoval Jr et al. [94], which pointed out that covariance matrix cleaning may actually lead to the worst portfolio performances in periods of high volatility.

Regarding the principal components of the covariance matrices and the dominance of the top eigenvalue discussed by the literature, the results showed that for all models and markets, the first eigenvalue of the covariance matrix was much bigger than the theoretical bound $\lambda_{max}$, which is consistent with the stylized facts discussed in Section 2. Moreover, for the vast majority of the cases (44 out of 54), the single top eigenvalue $\lambda^{top}$ contained more than 25% of all the variance. This result is consistent with the finding of previous similar works stated in the literature review section (Sensoy et al. [52] and others): the fact that a single principal component concentrated over 25% of the information is evidence that it captures the systematic risk, the very slice of the risk which cannot be diversified—in other words, the share of the risk that persists, regardless of the weight allocation. The results persisted for the eigenvalues above the upper bound of Equation (4): in more than half of the cases (31 out of 54), the “non-noisy” eigenvalues represented more than half of the total variance. The concentration of information in non-noisy eigenvalues in polynomial Kernels was weaker than the linear covariance matrices, while for the Gaussian Kernel the percentage of variance retained was even larger—around 70% of the total variance for all seven markets.

Finally, the columns $p_{Sharpe}$ and $p_{Sortino}$ show the statistical significance of the improvement of Sharpe and Sortino ratios brought about by the introduction of noise filtering based on the Random Matrix Theory. The results indicate that, while in some cases the noise filtering worked very well, in other cases it actually worsened the portfolio’s performances. Therefore, there is evidence that better portfolios can be achieved by eliminating the “noisy eigenvalues”, but the upper bound given by Equation (4) may be classifying actually informative principal components as “noise”. Especially concerning Kernel covariance matrices, the effects of the eigenvalues cleaning seemed unstable, working well in some cases and very bad in others, suggesting that the dynamics of the eigenvalues in nonlinear covariance matrices follow a different dynamic than linear ones, and the information that is considered to be “noise” for linear estimates can actually be informative in nonlinear domains. At the usual 95% confidence level, evidences of statistical superiority of filtered covariance matrices was present in 25 out of 54 cases for the Sharpe ratio (rejection of null hypothesis in (19)) and 26 out of 54 for the Sortino ratio (rejection of null hypothesis in (20)). The markets in which more models showed significant improvement using the Random Matrix Theory were the French and the German; on the other hand, again, for a less efficient financial market like the Brazilian one, the elimination of noisy eigenvalues yielded the worst performances (the profitability of all portfolios actually dropped), again consistent with the finding of Sandoval Jr et al. [94].

## 6. Conclusions

In this paper, the effectiveness of introducing nonlinear interactions to the covariance matrix estimation and its noise filtering using the Random Matrix Theory was tested with daily data from seven different financial markets. We tested eight estimators for the covariance matrix and evaluated the statistical significance of the noise-filtering improvement on portfolio performance. While the cleaning of noisy eigenvalues did not show significant improvements in every analyzed market, the out-of-sample Sharpe and Sortino ratios of the portfolios were significantly improved for almost half of all tested cases. The findings of this paper can potentially aid the investment decision for scholars and financial market participants, as well as providing both theoretical and empirical tools for the construction of more profitable and less risky trading strategies, as well as exploring potential weaknesses of traditional linear methods of covariance estimation.

We also tested the introduction of different degrees of nonlinearities to the covariance matrices by means of Kernel functions, with varied results: while in some cases, the Kernel approach managed to get better results, for others the addition yielded a much worse performance, indicating that the use of Kernels represent a high boost of the models’ complexity levels, which are not always compensated by better asset allocations, even when part of the said additional complexity is filtered out. This implies that the noise introduced by nonlinear features can surpass the additional predictive power which they aggregate to the Markowitz model. To further investigate this result, future developments include testing other Kernel functions besides the polynomial and the Gaussian to investigate whether alternative frameworks of nonlinear dependence can show better results. For instance, the results shown by different classes of Kernel functions [95] may fit better into the financial markets’ stylized facts and reveal underlying patterns based on the Kernel’s definition. Tuning the hyperparameters for each Kernel can also influence the model’s performance decisively.

Although the past performance of a financial asset does not determine its future performance, in this paper we kept in the dataset only the assets that composed of the seven financial indexes during the whole period between 2000 and 2018, thus not considering the possible survivorship bias in the choice of the assets which can affect the model’s implications [96]. As for future advancements, the difference between the “surviving” assets from the others can be analyzed as well. Other potential improvements include the replication of the analysis for other financial indexes or markets and other time periods, incorporation of transaction costs, and comparison with other portfolio selection models apart from Markowitz’s.

This paper focused on the introduction on nonlinear interactions to the covariance matrix estimation. Thus, a limitation was the choice of the filtering methods, as the replacement procedure that we adopted was not the only one that the literature on the Random Matrix Theory recommends. Alternative filtering methods documented by studies like Guhr and Kälber [97] and Daly et al. [98], such as exponential weighting and Krzanowski stability maximization, may allow for better modeling of underlying patterns of financial covariance structures and also lead to better portfolio allocations, such that the application of those methods and comparison to our proposed methods can be a subject of future research in this agenda.

## Figures and Tables

**Table 1:** Summary results for assets of the NASDAQ-100 Index: CR is the cumulative return of the optimal portfolio in the out-of-sample period; $\lambda^{*}$ is the number of non-noisy eigenvalues of the respective covariance matrix; $\lambda_{variance}^{*}\left(\%\right)$ is the percentage of variance explained by the non-noisy eigenvalues; $\lambda^{top}$ is the value of the top eigenvalue; $\lambda_{variance}^{top}\left(\%\right)$ is the percentage of variance that the top eigenvalue is responsible for; $p_{Sharpe}$ is the *p*-value of the hypothesis test (19); and $p_{Sortino}$ is the *p*-value of the hypothesis test (20).

Covariance	Method	CR (%)	Sharpe	Sortino	$\lambda^{*}$	$\lambda_{\mathrm{variance}}^{*}\left(\%\right)$	$\lambda^{\mathrm{top}}$	$\lambda_{\mathrm{variance}}^{\mathrm{top}}\left(\%\right)$	$p_{\mathrm{Sharpe}}$	$p_{\mathrm{Sortino}}$
Matrix			Ratio	Ratio						
Non-filtered	Pearson	22.3297	0.3252	0.4439						
	MCD	19.1094	0.2713	0.3690						
	RMCD	18.6733	0.2632	0.3574						
	OGK	21.2332	0.3037	0.4138						
	K_POLY2	28.7582	0.3808	0.5144						
	K_POLY3	28.7561	0.3884	0.5253						
	K_POLY4	29.7912	0.4108	0.5561						
	K_GAUSS	13.7226	0.1703	0.2304						
Filtered	Pearson	18.9984	0.2834	0.3847	5	45.38%	20.0680	33.33%	0.9432	0.9874
	MCD	23.9648	0.3595	0.4924	5	51.1%	24.6837	40.99%	0.0004	<$10^{-4}$
	RMCD	23.4073	0.3459	0.4730	5	51.19%	24.6470	40.93%	0.0011	<$10^{-4}$
	OGK	23.6193	0.3512	0.4809	5	49.53%	23.7152	39.39%	0.0382	0.0061
	K_POLY2	15.831	0.2218	0.3015	5	38.24%	16.1131	26.76%	>0.9999	>0.9999
	K_POLY3	16.7263	0.2496	0.3389	5	26.23%	9.2748	15.4%	>0.9999	>0.9999
	K_POLY4	16.186	0.2417	0.3283	5	19.29%	5.7377	9.53%	>0.9999	>0.9999
	K_GAUSS	21.823	0.2496	0.3435	5	67.89%	24.9393	41.42%	0.0015	<$10^{-4}$

**Table 2:** Summary results for assets of the FTSE 100 Index: CR is the cumulative return of the optimal portfolio in the out-of-sample period; $\lambda^{*}$ is the number of non-noisy eigenvalues of the respective covariance matrix; $\lambda_{variance}^{*}\left(\%\right)$ is the percentage of variance explained by the non-noisy eigenvalues; $\lambda^{top}$ is the value of the top eigenvalue; $\lambda_{variance}^{top}\left(\%\right)$ is the percentage of variance that the top eigenvalue is responsible for; $p_{Sharpe}$ is the *p*-value of the hypothesis test (19); and $p_{Sortino}$ is the *p*-value of the hypothesis test (20).

Covariance Matrix	Method	CR (%)	Sharpe Ratio	Sortino Ratio	$\lambda^{*}$	$\lambda_{\mathrm{variance}}^{*}\left(\%\right)$	$\lambda^{\mathrm{top}}$	$\lambda_{\mathrm{variance}}^{\mathrm{top}}\left(\%\right)$	$p_{\mathrm{Sharpe}}$	$p_{\mathrm{Sortino}}$
Non-filtered	Pearson	−16.8525	−0.2443	−0.3236						
	MCD	−23.9938	−0.3252	−0.4203						
	RMCD	−24.2595	−0.3272	−0.4223						
	OGK	−23.5119	−0.3223	−0.4178						
	K_POLY2	−2.4443	−0.0377	−0.0483						
	K_POLY3	−3.0975	−0.0453	−0.0575						
	K_POLY4	−3.1496	−0.0462	−0.0583						
	K_GAUSS	−5.4357	−0.0772	−0.1022						
Filtered	Pearson	−15.1099	−0.2246	−0.2986	6	52.52%	22.7137	38.24%	0.0222	0.0051
	MCD	−22.5761	−0.3148	−0.4096	6	55.87%	25.6111	43.12%	0.1547	0.1491
	RMCD	−22.8926	−0.3178	−0.4131	6	56.27%	25.8719	43.55%	0.1813	0.1852
	OGK	−22.3237	−0.3142	−0.4104	6	55.15%	25.2449	42.5%	0.2137	0.2326
	K_POLY2	−13.825	−0.2029	−0.2711	5	47.84%	21.2488	35.77%	>0.9999	>0.9999
	K_POLY3	−12.2619	−0.1812	−0.2413	7	38.27%	13.3597	22.49%	>0.9999	>0.9999
	K_POLY4	−10.2092	−0.1539	−0.2028	9	33.23%	8.6809	14.61%	>0.9999	>0.9999
	K_GAUSS	6.9977	0.0657	0.0908	7	75.37%	25.9374	43.66%	<$10^{-4}$	<$10^{-4}$

**Table 3:** Summary results for assets of the CAC 40 Index: CR is the cumulative return of the optimal portfolio in the out-of-sample period; $\lambda^{*}$ is the number of non-noisy eigenvalues of the respective covariance matrix; $\lambda_{variance}^{*}\left(\%\right)$ is the percentage of variance explained by the non-noisy eigenvalues; $\lambda^{top}$ is the value of the top eigenvalue; $\lambda_{variance}^{top}\left(\%\right)$ is the percentage of variance that the top eigenvalue is responsible for; $p_{Sharpe}$ is the *p*-value of the hypothesis test (19); and $p_{Sortino}$ is the *p*-value of the hypothesis test (20).

Covariance Matrix	Method	CR (%)	Sharpe Ratio	Sortino Ratio	$\lambda^{*}$	$\lambda_{\mathrm{variance}}^{*}\left(\%\right)$	$\lambda^{\mathrm{top}}$	$\lambda_{\mathrm{variance}}^{\mathrm{top}}\left(\%\right)$	$p_{\mathrm{Sharpe}}$	$p_{\mathrm{Sortino}}$
Non-filtered	Pearson	16.2333	0.2015	0.2882						
	MCD	17.2074	0.2182	0.3117						
	RMCD	17.4111	0.2216	0.3165						
	OGK	17.6784	0.2264	0.3235						
	K_POLY2	11.8756	0.1423	0.1963						
	K_POLY3	10.6055	0.1311	0.1793						
	K_POLY4	9.5146	0.1188	0.1614						
	K_GAUSS	12.3998	0.1348	0.1928						
Filtered	Pearson	17.4651	0.2238	0.3199	3	56.82%	14.1697	48.52%	0.0147	0.0010
	MCD	18.9068	0.2475	0.3533	2	58.57%	15.9837	54.73%	0.0022	<$10^{-4}$
	RMCD	19.0796	0.2504	0.3575	2	58.38%	15.9013	54.45%	0.0019	<$10^{-4}$
	OGK	18.6063	0.2423	0.3461	2	56.89%	15.4144	52.78%	0.0578	0.0126
	K_POLY2	16.5982	0.2076	0.2969	3	51.5%	12.5296	42.9%	<$10^{-4}$	<$10^{-4}$
	K_POLY3	17.8811	0.2289	0.3274	4	42.31%	8.6342	29.57%	<$10^{-4}$	<$10^{-4}$
	K_POLY4	17.7003	0.2333	0.3311	4	33.88%	6.1270	20.98%	<$10^{-4}$	<$10^{-4}$
	K_GAUSS	11.5206	0.1228	0.1757	4	78.74%	16.0889	55.09%	0.8828	0.9549

**Table 4:** Summary results for assets of the DAX-30 Index: CR is the cumulative return of the optimal portfolio in the out-of-sample period; $\lambda^{*}$ is the number of non-noisy eigenvalues of the respective covariance matrix; $\lambda_{variance}^{*}\left(\%\right)$ is the percentage of variance explained by the non-noisy eigenvalues; $\lambda^{top}$ is the value of the top eigenvalue; $\lambda_{variance}^{top}\left(\%\right)$ is the percentage of variance that the top eigenvalue is responsible for; $p_{Sharpe}$ is the *p*-value of the hypothesis test (19); and $p_{Sortino}$ is the *p*-value of the hypothesis test (20).

Covariance Matrix	Method	CR (%)	Sharpe Ratio	Sortino Ratio	$\lambda^{*}$	$\lambda_{\mathrm{variance}}^{*}\left(\%\right)$	$\lambda^{\mathrm{top}}$	$\lambda_{\mathrm{variance}}^{\mathrm{top}}\left(\%\right)$	$p_{\mathrm{Sharpe}}$	$p_{\mathrm{Sortino}}$
Non-filtered	Pearson	6.3447	0.0772	0.1027						
	MCD	−1.5643	−0.0315	−0.0414						
	RMCD	−0.378	−0.0161	−0.0212						
	OGK	5.3011	0.0615	0.0815						
	K_POLY2	−4.6104	−0.0733	−0.0949						
	K_POLY3	−0.6555	−0.0204	−0.0265						
	K_POLY4	1.7874	0.0131	0.0171						
	K_GAUSS	−10.2399	−0.1311	−0.1720						
Filtered	Pearson	10.2332	0.1346	0.1796	3	55.24%	11.0402	46.1%	0.0014	<$10^{-4}$
	MCD	7.0445	0.0866	0.1149	2	58.39%	12.8292	53.57%	<$10^{-4}$	<$10^{-4}$
	RMCD	7.5928	0.0942	0.1254	2	58.88%	12.9601	54.11%	<$10^{-4}$	<$10^{-4}$
	OGK	9.8916	0.1286	0.1715	2	56.32%	12.3346	51.5%	0.0003	<$10^{-4}$
	K_POLY2	4.3642	0.0484	0.0640	2	46.78%	9.9835	41.69%	<$10^{-4}$	<$10^{-4}$
	K_POLY3	6.7303	0.0830	0.1099	3	38.77%	6.9275	28.93%	<$10^{-4}$	<$10^{-4}$
	K_POLY4	9.7678	0.1297	0.1717	4	35.17%	5.0114	20.93%	<$10^{-4}$	<$10^{-4}$
	K_GAUSS	−18.5834	−0.2365	−0.3050	2	71.04%	13.7234	57.3%	>0.9999	>0.9999

**Table 5:** Summary results for assets of the NIKKEI 225 Index: CR is the cumulative return of the optimal portfolio in the out-of-sample period; $\lambda^{*}$ is the number of non-noisy eigenvalues of the respective covariance matrix; $\lambda_{variance}^{*}\left(\%\right)$ is the percentage of variance explained by the non-noisy eigenvalues; $\lambda^{top}$ is the value of the top eigenvalue; $\lambda_{variance}^{top}\left(\%\right)$ is the percentage of variance that the top eigenvalue is responsible for; $p_{Sharpe}$ is the *p*-value of the hypothesis test (19); and $p_{Sortino}$ is the *p*-value of the hypothesis test (20).

Covariance Matrix	Method	CR (%)	Sharpe Ratio	Sortino Ratio	$\lambda^{*}$	$\lambda_{\mathrm{variance}}^{*}\left(\%\right)$	$\lambda^{\mathrm{top}}$	$\lambda_{\mathrm{variance}}^{\mathrm{top}}\left(\%\right)$	$p_{\mathrm{Sharpe}}$	$p_{\mathrm{Sortino}}$
Non-filtered	Pearson	19.0365	0.2104	0.2976						
	MCD	17.9163	0.1979	0.2791						
	RMCD	18.3996	0.1983	0.2803						
	OGK	17.833	0.1951	0.2757						
	K_POLY2	8.5753	0.0959	0.1325						
	K_POLY3	10.6699	0.1233	0.1700						
	K_POLY4	13.1313	0.1553	0.2145						
	K_GAUSS	14.5078	0.1586	0.2236						
Filtered	Pearson	19.4964	0.2231	0.3161	12	54.88%	57.4396	39.38%	0.1347	0.0540
	MCD	18.266	0.2025	0.2855	11	57.24%	63.4158	43.48%	0.3498	0.2938
	RMCD	19.0273	0.2119	0.2987	12	58.83%	65.3846	44.83%	0.1235	0.0591
	OGK	19.0061	0.2142	0.3023	11	56.5%	62.0915	42.57%	0.0501	0.0111
	K_POLY2	15.1032	0.1637	0.2314	11	47.71%	49.6729	34.06%	<$10^{-4}$	<$10^{-4}$
	K_POLY3	16.8414	0.1890	0.2661	13	35.62%	30.0585	20.61%	<$10^{-4}$	<$10^{-4}$
	K_POLY4	18.2374	0.2090	0.2943	14	27.44%	18.6121	12.76%	<$10^{-4}$	<$10^{-4}$
	K_GAUSS	12.6904	0.1385	0.1953	15	72.24%	42.7789	29.33%	0.9570	0.9923

**Table 6:** Summary results for assets of the SSE 180 Index: CR is the cumulative return of the optimal portfolio in the out-of-sample period; $\lambda^{*}$ is the number of non-noisy eigenvalues of the respective covariance matrix; $\lambda_{variance}^{*}\left(\%\right)$ is the percentage of variance explained by the non-noisy eigenvalues; $\lambda^{top}$ is the value of the top eigenvalue; $\lambda_{variance}^{top}\left(\%\right)$ is the percentage of variance that the top eigenvalue is responsible for; $p_{Sharpe}$ is the *p*-value of the hypothesis test (19); and $p_{Sortino}$ is the *p*-value of the hypothesis test (20).

Covariance Matrix	Method	CR (%)	Sharpe Ratio	Sortino Ratio	$\lambda^{*}$	$\lambda_{\mathrm{variance}}^{*}\left(\%\right)$	$\lambda^{\mathrm{top}}$	$\lambda_{\mathrm{variance}}^{\mathrm{top}}\left(\%\right)$	$p_{\mathrm{Sharpe}}$	$p_{\mathrm{Sortino}}$
Non-filtered	Pearson	−24.4861	−0.2945	−0.3765						
	MCD	−18.4543	−0.2139	−0.2762						
	RMCD	−20.8369	−0.2393	−0.3073						
	OGK	−22.9376	−0.2617	−0.3364						
	K_POLY2	−36.7953	−0.3531	−0.4459						
	K_POLY3	−35.2879	−0.3460	−0.4335						
	K_POLY4	−34.3716	−0.3422	−0.4258						
	K_GAUSS	−33.6337	−0.3735	−0.4744						
Filtered	Pearson	−21.0991	−0.2587	−0.3308	11	50.96%	56.5957	38.99%	0.0011	<$10^{-4}$
	MCD	−25.1805	−0.2913	−0.3724	11	49.85%	54.7101	37.69%	>0.9999	>0.9999
	RMCD	−20.685	−0.2379	−0.3053	11	50.78%	56.5502	38.96%	0.4543	0.4344
	OGK	−21.7307	−0.2520	−0.3235	11	48.66%	52.5361	36.2%	0.2154	0.1482
	K_POLY2	−26.5935	−0.3140	−0.3978	12	41.25%	42.7236	29.44%	0.0007	<$10^{-4}$
	K_POLY3	−28.6612	−0.3292	−0.4140	13	28.83%	24.2135	16.68%	0.0870	0.0565
	K_POLY4	−28.9269	−0.3338	−0.4186	12	20.18%	14.1161	9.73%	0.2469	0.2801
	K_GAUSS	−38.4531	−0.4102	−0.5175	12	69.52%	60.1106	41.42%	0.9986	0.9998

**Table 7:** Summary results for assets of Bovespa Index: CR is the cumulative return of the optimal portfolio in the out-of-sample period; $\lambda^{*}$ is the number of non-noisy eigenvalues of the respective covariance matrix; $\lambda_{variance}^{*}\left(\%\right)$ is the percentage of variance explained by the non-noisy eigenvalues; $\lambda^{top}$ is the value of the top eigenvalue; $\lambda_{variance}^{top}\left(\%\right)$ is the percentage of variance that the top eigenvalue is responsible for; $p_{Sharpe}$ is the *p*-value of the hypothesis test (19); and $p_{Sortino}$ is the *p*-value of the hypothesis test (20).

Covariance Matrix	Method	CR (%)	Sharpe Ratio	Sortino Ratio	$\lambda^{*}$	$\lambda_{\mathrm{variance}}^{*}\left(\%\right)$	$\lambda^{\mathrm{top}}$	$\lambda_{\mathrm{variance}}^{\mathrm{top}}\left(\%\right)$	$p_{\mathrm{Sharpe}}$	$p_{\mathrm{Sortino}}$
Non-filtered	Pearson	9.3348	0.0636	0.0871						
	MCD	3.4975	0.0206	0.0280						
	RMCD	1.8602	0.0079	0.0107						
	OGK	3.0337	0.0167	0.0227						
	K_POLY2	15.2198	0.1127	0.1521						
	K_POLY3	16.2334	0.1184	0.1594						
	K_POLY4	16.6977	0.1194	0.1605						
	K_GAUSS	32.0362	0.1934	0.2657						
Filtered	Pearson	−3.5439	−0.0334	−0.0453	2	58.59%	13.5231	54.46%	>0.9999	>0.9999
	MCD	−3.8358	−0.0364	−0.0492	2	55.01%	12.5411	50.51%	0.9994	>0.9999
	RMCD	−1.6626	−0.0191	−0.0258	2	54.11%	12.2963	49.52%	0.9329	0.9787
	OGK	−4.5348	−0.0412	−0.0557	2	54.81%	12.5097	50.38%	0.9994	>0.9999
	K_POLY2	3.7777	0.0217	0.0296	2	47.88%	10.6994	43.09%	>0.9999	>0.9999
	K_POLY3	−4.0389	−0.0370	−0.0499	4	43.39%	7.3663	29.67%	>0.9999	>0.9999
	K_POLY4	−9.6085	−0.0809	−0.1087	4	35.63%	5.2703	21.23%	>0.9999	>0.9999
	K_GAUSS	31.7689	0.1916	0.2631	2	77.51%	16.0176	64.51%	0.5383	0.5568
